# Supplementary material for: Are carbon emissions trading and green financial instruments synergistic? -Comprehensive quantitative research based on content analysis
Source: PLoS One. 2024 Mar 7;19(3):e0298601. doi: 10.1371/journal.pone.0298601 (PMC10919601; doi:10.1371/journal.pone.0298601)
Supplement: S1 Data — (ZIP) [file pone.0298601.s001.zip › Data/地级市Z_F工作报告.zip!/║╙─╧/σo╤⌠╩╨/└┤╫╘╣1⁄2╓┌║┼í╛┬φ┐╦╩2╛▌═°í┐_2016.docx]

各位代表：

现在，我代表市人民政府，向大会报告工作，请予审议，并请各位政协委员和其他列席人士提出意见。

一、2015年及“十二五”工作回顾

2015年是我市致力新赶超、开拓新境界、赢得新发展的重要一年，是“十二五”规划的收官之年。一年来，在省委、省政府和市委的坚强领导下，在市人大、市政协的监督支持下，市政府认真贯彻党的十八大和十八届三中、四中、五中全会精神和习近平总书记系列重要讲话精神，面对复杂严峻的经济形势和艰巨繁重的发展任务，主动适应经济发展新常态，保态势、创优势，迎难而上，拼搏进取，实现了经济总量、人均数量、发展质量“三量齐升”，较好完成了市七届人大二次会议确定的目标任务，濮阳新一轮赶超发展迈出了坚实步伐。

（一）赶超发展势头强劲。面对经济下行压力，精准施策，推出了促进经济平稳健康发展30条、稳增长保态势27个重大专项等系列政策措施，打出了一套组合拳，实现了速度质量双提升。2015年，全市生产总值完成1333.64亿元，增长9.5%，增速居全省第2位；第三产业完成增加值421.65亿元，增长12.9%，居全省第1位；规模以上工业增加值764.14亿元，增长9.3%，居全省第9位；社会消费品零售总额470.68亿元，增长13.1%，居全省第3位；一般公共预算收入78.99亿元，增长12.2%，居全省第4位，税收占比74.3%，居全省第2位；一般公共预算支出212.32亿元，增长16.7%，居全省第6位。

（二）产业结构优化升级。产业结构日趋合理，三次产业比例达到11.8：56.6：31.6，三产占比首破30%。

工业经济增量提质。深入实施工业强市战略“131”工程，新增规模以上工业企业73家，达到987家。地方工业快速发展，增速高出中央和省属企业30.6个百分点；增加值占比87.1%，较上年提高5.5个百分点。石油和化工产业主营业务收入首次突破1000亿元。高新技术产业和战略性新兴产业快速发展，增速高出工业平均水平12.7个百分点，对规上工业增加值的贡献率达到59.5%。

第三产业增长迅速。第三产业首次实现两位数增长，成为拉动经济增长的重要力量。全年完成投资534.82亿元，增长33.1%，高出全市投资增速16.1个百分点。新增规模以上服务业单位549家。金融业快速发展，新增贷款82亿元，增长17.8%，增速居全省第3位。电商企业达到155家，新兴业态增势迅猛，商贸物流、文化旅游业快速发展。

农业发展迈出新步伐。抓好高标准粮田“百千万”工程，新建高标准粮田40.7万亩，粮食总产达到271.8万吨，实现“十二连增”。流转土地114.2万亩，新增现代农业园区20个、新增标准化养殖场50个。市级以上农业产业化龙头企业达到250家，省级以上达到32家。农民专业合作社较上年增加215家。农业生产机械化率81%。植树2千余万株，森林覆盖率达到25%。

（三）开放招商实现突破。创新招商机制，突出招商重点，大招商、招大商，成果丰硕。富士康正一特殊材料产业园开工建设，实现了引进世界500强企业的零突破。上海金山濮阳精细化工产业园成功签约，实现了集群引进的新突破。森源新能源环卫车辆、海王药械阳光集中配送中心等30个重大招商项目落户濮阳，与中建材、德力西、闻泰集团、昊华骏化、保利协鑫、东方雨虹、上海电气、建业等50家龙头企业签订了合作协议。全年签约亿元以上项目387个，新开工亿元以上项目358个，其中10亿元以上项目31个。利用省外资金194.3亿元，增长10%，增速居全省第1位；利用境外资金5.75亿美元，增长18.1%，居全省第1位。

开放合作不断深化。濮阳商检办事处业务不断扩大，我市被列为全省首批检商“两证合一”改革试点城市。在濮设立海关分支机构工作加快推进。外经外贸工作取得新成效，全市完成出口总额7.05亿美元，增长9.3%，服务贸易出口1.78亿美元，居全省第4位。我市被命名为“河南省杂技文化出口基地”。成功召开了油地高层领导联席会议第四次会议，谋划推进了一批重大基础设施和产业项目，油地融合发展迈出新步伐。

（四）发展优势持续积累。办成和推进了28项事关全局发展的大事要事。实施省市重点项目318个，完成投资969.8亿元。全市固定资产投资完成1305.28亿元，增长17%。

综合交通体系加快建设。郑濮济高铁前期工作正式启动，晋豫鲁铁路即将开通客运，濮阳至信阳潢川铁路纳入省规划。台辉高速豫鲁界至范县段开工奠基，南林高速东延、德商高速范县段建成通车，实现了与山东高速公路网的对接。加快干线公路升级改造，全市一级公路总里程达到243公里，新建改建农村公路550公里。

资源能源配置能力持续提升。石油、天然气资源进一步聚集。投资286亿元的文23战略储气库启动先导工程，中原油田石化总厂扩能改造、日濮洛输油管线工程取得新进展，濮阳至开封、至邯郸等输气管道建成投用。丰利石化丙烯联产芳烃项目投产试运行。投资59.6亿元的豫能2×60万千瓦机组项目进展顺利。南水北调濮阳配套工程建成通水。总投资44.78亿元的引黄入冀补淀工程濮阳段开工建设。

产业承载力不断增强。产业集聚区完成基础设施投资85.26亿元，增长31%。综合服务中心实现全覆盖。国家电光源质量检测中心、中科院光电技术联合实验室等一批检测研发平台建成投用。濮阳市化工产业集聚区获省批复，拓展了发展空间。商务中心区和特色商业区完成投资159.5亿元，入驻企业601家，新增101家。华龙区特色商业区荣获“河南省特色商业区建设金星奖”。

（五）改革创新成效显著。完成了市、县政府机构改革。深化了行政审批制度改革，承接下放行政审批事项49项。推行了“两个清单”管理制度。降低工商注册门槛，实行“三证合一”，全年新增各类市场主体5.41万户，增长27.44%。深化投融资体制改革，整合组建了市投资集团，总资产达到130亿元。创新投资模式，争取专项基金，发行项目收益债，推进了台辉高速公路濮阳段建设。设立助保贷资金、过桥资金，有效缓解了企业融资难题。惠成电子在创业板上市，蔚林化工、盛源科技等5家企业在新三板挂牌交易。强化地方性债务管理，置换政府债务40亿元，节约资金2.2亿元。PPP项目建设取得新进展。农村集体土地和宅基地使用权确权颁证完成70%。完成了交通运输执法体制改革。事业单位分类改革、城镇化综合改革、文化体制改革、党政机关公务用车改革取得阶段性成果。国有企业、医药卫生、司法体制、公安、供销、机关事业单位养老保险等改革扎实推进。2015年，我市多项改革走在了全省前列，率先推出了“房地产新政”，实施的购房定向补贴、组织引导团购等创新性措施，去库存效果明显，得到国家、省的高度评价；率先启动财政涉企资金基金化改革，设立了省辖市首家产业投资基金；率先整合市县担保机构，成立了首家市级担保集团；率先在省辖市实现供水、热力资产证券化；率先开展财政存量资金盘活工作，盘活资金6亿元；率先完成了食品药品监管体制改革；率先对审批事项实行“多评合一”、网上审批，提高了审批效能。

创新能力不断增强。新增高新技术企业7家、省市节能减排科技创新示范企业10家，新建省、市级工程技术研究中心17家、市级重点实验室4家。沃森超高分子量聚乙烯技术与产能处全国领先地位，举办了高端论坛，建立了创新联盟。与清华大学、上海交大、同济大学等科研院校建立了合作关系。万人有效发明专利拥有量增长9个百分点，居全省第7位。新增专利技术产业化示范企业5家。成立了众创空间联盟。我市荣获河南省科技进步一等奖两项，居全省第1名。

（六）城乡发展协调推进。切实解决农业转移人口就业、社保、住房等问题，突出教育拉动，市县城区学校扩招2.38万人，带动近6万人进城。我市被列为国家新型城镇化综合试点城市。

加快构建现代城镇体系。市域中心城市建设取得新成效。发挥规划引领作用，综合提升建成区，加快发展示范区，加大“五网四厂两道”等基础设施建设，推进绿系水系、海绵城市、智慧城市建设，实施精细化管理，巩固了文明城市创建成果，通过了国家卫生城市复检，完善了城市功能，提高了城市品位。县域中心城市竞相发展，承载力、带动力明显提升。清丰县创建成为国家卫生县城、园林县城。范县创建成为国家卫生县城。濮阳县、南乐县、台前县创建成为省级卫生县城。小城镇建设取得新进展，濮阳县柳屯镇、文留镇、城关镇、范县濮城镇跻身全省百强乡镇，清丰县仙庄镇创建成为省级生态乡镇，濮阳县城关镇、柳屯镇跻身财政收入亿元镇行列。

美丽乡村建设取得新成效。按照“通、净、绿、亮、文”要求，完善垃圾收集、运输、处理机制，加强公共服务和文化设施建设，巩固提升了示范村整治成果，农村面貌大为改观。西辛庄、单拐、毛楼等20个村庄入选河南最美乡村。

（七）人民生活明显改善。全市财政用于民生的支出171.3亿元，占一般公共预算支出的80.7%，居全省第2位。十项重点民生工程全面完成。全市城镇居民人均可支配收入24928元，增长7.4%；农民人均可支配收入9790元，增长9.8%。

就业和社会保障体系不断完善。全市城镇新增就业7.3万人，新增农村劳动力转移就业3.3万人。发放小额担保贷款4.5亿元，支持2.3万人创业就业。企业退休人员基本养老金实现“十一连增”。市残疾人综合服务中心开工建设。完成了73所乡镇敬老院提升、300个农村互助幸福院建设工程。

脱贫攻坚迈出坚实步伐。实施精准扶贫，全年实现脱贫5.25万人。黄河滩区第一批居民迁建试点扎实推进。

社会事业加快发展。创建农村标准化学校197所，实施校安工程项目47个。新建改建城区学校6所。河南大学濮阳工学院实现招生。濮阳医专（筹）完成搬迁，市委党校新校区开工奠基。市眼科医院新址建成投用，新建改建乡镇卫生院21所、村卫生室629所。文化设施建设、文化下乡等文化惠民工程成效明显。全民健身活动蓬勃开展，我市被列为全国乒乓球三大训练基地之一。

生态环境不断改善。强化环境监管，实施“蓝天工程”、“碧水工程”，开展“环保风暴”、“排雷行动”，综合整治城市水系，整改排放不达标企业76家。节能减排完成省定目标。濮阳市国家生态文明先行示范区建设方案通过评审。我市荣获2015年创建生态文明标杆城市。

社会大局和谐稳定。安全生产监管力度不断加大，食品安全形势持续向好。切实做好信访稳定工作，平安濮阳、法治濮阳建设扎实推进，社会大局保持和谐稳定。我市政法机关综合执法满意度、公众安全感连续三年荣获全省“双第一”。我市被评为全省平安建设先进市。

（八）政府自身建设不断加强。坚持依法行政，完善责任、有序、高效的工作机制，行政效能不断提升。坚持民主科学决策，认真执行人大及其常委会决议和决定，支持政协参政议政，努力做好人大代表建议和政协委员提案办理工作。特别是对市人大代表提出的水系规划建设和水环境治理、城区农贸市场建设与管理等议案，市政协委员提出的实施创新驱动推动产业升级、大力发展电子商务等提案，政府高度重视，强力推进，取得了良好效果。广泛听取民主党派、工商联、无党派人士意见，支持工会、共青团、妇联等人民团体开展工作，自觉接受各方监督。开展“三严三实”专题教育，深入落实中央八项规定精神，认真落实“两个责任”。强化行政监察、审计监督、投资监管，开展懒政怠政为官不为专项治理，严肃查处违纪违法案件，保障财政资金使用效益，着力营造为民、务实、清廉的政务环境。

国防教育、双拥工作扎实开展，驻濮解放军和武警部队在支持地方发展、维护稳定方面做出了积极贡献。各民主党派、市工商联和无党派人士，站位全局，积极作为，深化“同心”实践，有力促进了濮阳发展。统计、民族宗教、发展研究、外事侨务、机关事务、驻外联络、公积金管理、残疾人工作、气象、防震减灾、人防、史志、档案等工作都取得了新成绩。

当前的濮阳，城乡更加靓丽，人民更加自信，前景更加广阔！

各位代表！

2015年目标任务的完成，标志着我市“十二五”规划的圆满收官，濮阳发展又站上了新的历史起点。五年来，我们深入开展党的群众路线教育实践活动和“三严三实”专题教育，持续开展“一创双优”活动，打破了沉闷、焕发了生机，顺应了大势、经受了挑战，矢志不渝、拼搏进取，取得了一系列标志性突破，开启了赶超发展的新征程，步入了科学发展的新阶段，做出了无愧于人民的新业绩。

一组数据印证着“十二五”时期的铿锵步履。

——经济发力提速，实力大幅跃升。2015年全市生产总值是2010年的1.7倍，年均增长11.3%；人均生产总值36822元，是2010年的1.7倍；一般公共预算收入、支出分别是2010年的2.6倍、2.4倍；金融机构存款余额1165.31亿元、贷款余额543.35亿元，分别是2010年的2倍、2.3倍。

——产业不断壮大，结构明显优化。粮食生产实现“十二连增”。2015年规模以上工业增加值是2010年的1.9倍，年均增长14.3%；三次产业比例由2010年的13.8：66.5：19.7调整为11.8：56.6：31.6；服务业成为新的增长点，增加值是2010年的1.7倍，年均增长11%。

——优势持续积累，发展更有底气。2015年全社会固定资产投资是2010年的2.5倍，年均增长20.1%；办成了一大批事关濮阳长远发展的大事要事，积蓄了势能，拓展了空间。

——城乡面貌巨变，人民生活殷实。建成区综合提升成效显著，示范区建设取得阶段性成果，县城、小城镇竞相发展，美丽乡村建设扎实推进，成功创建为全国文明城市，常住人口城镇化率由2010年的31.5%提高到40.35%。2015年城镇居民、农民人均可支配收入分别是2010年的1.7倍、1.9倍，年均分别增长11%和13.8%。各项社会事业全面发展，社会大局和谐稳定。

各位代表！

这些成绩是省委、省政府和市委正确领导的结果，是市人大、市政协大力支持的结果，凝聚着全市人民的心血和汗水。在此，我代表市政府，向工作在各条战线的工人、农民、知识分子、干部，人民解放军指战员、武警官兵、公安民警以及关心、支持濮阳发展的各界人士致以崇高的敬意和衷心的感谢！

“十二五”的五年，是濮阳发展历程中具有里程碑意义的五年。五年的不懈奋斗，五年的艰辛探索，深化了我们对科学发展、赶超发展的认识，获得了宝贵的经验。

一要解放思想，敢想敢干。持续开展“一创双优”活动，强化干部培训教育，激活了思想、转变了站位，催生了赶超发展的新理念、新思路、新举措，打开了空间、走出了困局，开启了赶超发展的新阶段，拓展了科学发展的新境界。实践证明，只有以解放思想为先导，才能引领我们的事业不断走向胜利。

二要遵循规律，科学谋划。面对复杂的外部环境和繁重的发展任务，市委、市政府审时度势，遵循发展规律，顺应群众期盼，确立了赶超发展的战略部署和总体思路，明确了新常态下“保持态势、创新优势、转型升级、赶超发展”主基调，闯出了一条欠发达地区跨越式发展的新路子。实践证明，只有遵循规律、切中肯綮，才能汇聚力量、实现赶超发展。

三要夯实基础，厚植优势。面对激烈的区域竞争，我们立足当前打基础，谋划长远创优势，办成了一批事关全局的大事要事，区位、资源、产业、环境等优势日益彰显，濮阳发展势头好、底气足，前景广阔。实践证明，只有不断创新优势，才能赢得发展主动权。

四要创新机制，激发干劲。濮阳赶超发展的良好局面，得益于我们建立健全了一套工作谋划、决策、推进、落实的有效机制，凝聚了发展合力，提高了执行力，形成了干事创业的生动局面。实践证明，制度机制是激发动力活力、推动工作落实、实现奋斗目标的坚强保障。

在肯定成绩、总结经验的同时，我们必须清醒忧患，正视存在的问题和短板。一方面，我市长期积累的体制机制和结构性矛盾依然存在。产业层次不高，创新能力不强，基础设施建设欠账较多，公共服务水平低，城镇化水平不高，脱贫攻坚任务艰巨，体制机制不活。另一方面，当前经济工作还面临着一些突出问题。受市场需求不足、产品价格下降和生产要素价格上升等因素影响，部分企业生产经营困难；受国际原油价格下跌冲击，油气采掘及装备制造等产业困难加剧；环境保护有待加强；政府职能转变还不到位，一些干部素质、能力、作风需要进一步强化等。对此，必须高度重视，认真解决。

二、“十三五”发展总体思路和目标任务

“十三五”时期是我市全面建成小康社会的决胜阶段，是保持态势、创新优势、转型升级、赶超发展的关键时期。制定和实施好我市“十三五”规划，描绘好未来五年发展蓝图，事关重大，影响长远。根据市委建议，市政府编制了“十三五”规划纲要（草案），已与纲要说明一起印发会议提请审议。

“十三五”时期经济社会发展的指导思想是：全面贯彻党的十八大和十八届三中、四中、五中全会精神，以马克思列宁主义、毛泽东思想、邓小平理论、“三个代表”重要思想、科学发展观为指导，深入贯彻习近平总书记系列重要讲话精神，以“四个全面”战略布局为总引领，以推进供给侧结构性改革为总抓手，以全面建成小康社会为总目标，以“坚持率先转型，实现三量齐升，进入全省省辖市第一方阵”为总要求，牢固树立创新、协调、绿色、开放、共享发展理念，着力改革开放，着力创新引领，着力优化结构，着力改善民生，全面建设富裕文明和谐美丽新濮阳。

主要预期目标是：生产总值年均增速达到8.5%以上，主要经济指标增速高于全省平均水平，确保“十三五”末全市生产总值和城乡居民人均收入比2010年翻一番以上；转型升级和创新驱动取得重大进展，产业迈向中高端水平，基本形成现代产业新体系；城乡协调发展取得显著成效，常住人口城镇化率达到56%；打赢脱贫攻坚战，确保现行国家标准下，农村贫困人口实现脱贫，贫困县全部摘帽；绿色低碳循环发展取得实质性进展，绿系水系基本成型，生态环境明显改善；社会事业全面进步，人民生活水平显著提升。到2020年，与全国全省同步全面建成小康社会，打造成为中原经济区与京津冀和山东半岛蓝色经济区对接的桥头堡、豫鲁冀三省交会区域性中心城市、全国重要的新型能源化工基地、全国重要的天然气输配中心、国家改革创新先行试验区、宜居宜业生态城市，基本建成创新开放、富裕文明、平安和谐、生态宜居的新濮阳。

实现上述目标，推动濮阳新一轮赶超发展，要把创新作为引领发展的第一动力，深化改革，创新驱动，加快产业向中高端方向发展，着力构建产业新体系；要突出协调发展，更加注重城乡一体化发展，统筹推动物质文明和精神文明建设，着力构筑均衡发展新格局；要突出绿色发展，更加注重环境保护和生态文明，着力建设美丽新家园；要突出开放发展，积极融入国家和区域重大发展战略，主动承接产业转移，着力拓展战略新空间；要突出共享发展，更加关注人民福祉，打赢脱贫攻坚战，着力创造幸福新生活。

各位代表！

蓝图已经绘就。宏伟的事业需要我们去奋斗，锦绣的前程等待我们去创造。我们坚信，只要全市上下凝心聚力、拼搏进取，我们的目标一定能够实现，濮阳的明天一定更加灿烂！

三、2016年发展目标和重点工作

2016年是实施“十三五”规划、全面建成小康社会决胜阶段的开局之年，做好今年的政府工作，责任重大，影响深远。当前，宏观经济形势依然复杂严峻，但经济总体向好的基本面没有改变。综合来看，濮阳未来发展机遇大于挑战，我们要主动实施供给侧结构性改革，融入国家、省发展战略，积极承接产业转移，加快基础设施建设，发挥好业已形成的综合优势，保持定力，闯关过坎，弯道超车，牢牢把握新一轮赶超发展主动权。

2016年我市经济社会发展的主要预期目标是：生产总值增长9%左右；规模以上工业增加值增长10%左右；一般公共预算收入增长9%以上；固定资产投资增长20%；社会消费品零售总额增长13%；外贸出口总额增长5.5%以上；实际利用外资增长10%；城镇居民和农民人均可支配收入分别增长8%、10%；居民消费价格指数涨幅控制在3%以内；节能减排实现省定目标。

做好今年的政府工作，要做到“五个必须”：必须抓住发展这个第一要务不放松，拼上去、过关口，扩投资、增消费，促进经济快速增长；必须抓住转型发展这条主线不放松，用好改革开放创新动力，去产能、去库存、去杠杆、降成本、补短板，重构产业新体系，实现蝶变升级；必须抓住大事要事这个关键不放松，上项目、夯基础，培育竞争新优势；必须抓住城市建设这个龙头不放松，打造区域性中心城市，增强吸引力、辐射力，拉动经济社会协调发展；必须抓住惠民生保稳定这一底线不放松，强化社会保障，实施脱贫攻坚，发展社会事业，实现共享发展。具体来讲，要抓好以下十个方面工作：

（一）提高供给质量，实现工业转型升级新突破

深入实施工业强市战略“131”工程，坚持加减乘除并举，改造传统产业、培育新兴产业并重，推动我市工业向中高端水平迈进。2016年，实施重点工业项目113个，年内完成投资304.35亿元。

改造提升传统产业。化工产业，要以提升价值为核心，以产业链重构为着力点，加快升级再造。上游以聚集资源为重点，加快推进丰利石化丙烯联产芳烃、班德路化学萜烯树脂等项目达产达效，推进中原大化气化岛、中原乙烯扩能改造等龙头项目取得新进展。下游依托基础化工优势，重点发展功能高分子材料、电子化工材料、高性能工程塑料、特种合成橡胶、3D打印材料等，重点推进惠成电子、沃森科技、迈奇科技、蔚林化工等企业发展壮大，加快推进富士康正一特殊材料、上海金山濮阳精细化工产业园等项目建设，加快建设濮阳市化工产业集聚区，着力打造重要的新型化工和化工新材料产业基地。

装备制造业，以战略合作、创新驱动为路径，拓展领域，提升发展层次。抓好中原特车与闻泰集团、中原总机厂与森源重工等合作项目的推进，加快中福矿山机械制造、中科润石油环保设备制造等项目建设，开拓能源装备、非常规油气装备市场。规划建设濮阳电气制造产业园，推进德力西电气、汉阳电子、长润电子等项目建设。

家具制造业，实施品牌战略，适应消费升级趋势，采取个性化、定制式生产模式，加快产业升级。重点推进全友家私、美松爱家、浙豫木业产业园等项目建设。

羽绒制品和食品加工业，以构建全产业链为重点，采取“基地+加工+市场”模式做大规模、提升质量。推进鹏达羽绒、兴隆羽绒、物华食品、春韵休闲食品、木伦河冷饮扩建等项目建设。

培育发展战略性新兴产业。培育与引进并重，重点发展新能源、新材料、节能环保、生物医药、高端装备制造等产业，形成濮阳未来发展的战略支撑。

在发展化工新材料的同时，大力发展生物基材料，充分利用我市作为国家生物基材料产业集群建设示范市的机遇，实施好永乐生物L-乳酸、龙都天仁聚乳酸树脂等项目，完善产业链条，不断壮大产业规模，着力打造重要的生物基材料基地。以濮耐股份为龙头，推动耐火材料向高端、节能、绿色方向发展。加快推进东方雨虹防水材料等项目。

大力发展新能源、清洁能源及相关产业。支持天能集团提质扩能，以此为依托，招大引强，重点推进望联新能源汽车制造等项目，构建从新能源电池到新能源汽车制造产业链。要做好天然气利用这篇大文章，以绿能融创LNG、LMG为龙头，大力发展清洁能源和清洁能源汽车。密切关注天然气制烯烃等前沿动态，谋划引进项目，构建产业链，提高附加值，将资源优势转化为经济优势。推进与昊华骏化集团开展战略合作，建设醋酸制乙醇等项目。推进华能光伏发电、天润清丰风电场、中昊光伏发电、中石化新星地热综合利用、洪峰新能源地热发电等项目，加快地热综合开发利用。同时，着力培育节能环保、生物医药等产业。

中原油田在我市产业发展中占有举足轻重的地位。要坚持完善油地战略合作机制，巩固提升油地战略合作成果，围绕产业转型升级，强化产业互动，共同推进中原石化总厂扩能改造等重大项目，实现油地合作向纵深发展。

充分发挥企业在转型中的主体作用。一是帮助企业降低成本。认真落实国家、省一系列政策措施，结合我市实际，研究下调政府性担保公司保费率，清理行政性收费，规范中介服务收费等。要发挥企业金融服务团作用，切实帮助企业解决融资难、融资贵问题。二是做好精准服务。加强工业经济运行监测预警，实施精准服务，搞好“四个对接”，保障企业正常生产经营。三是实施品牌带动战略，强化质量管理，创建全国质量强市示范城市。四是创新政策支持方式。采取基金参与、购买服务、股权投资等方式，支持企业发展和优质项目建设。

（二）注重城乡协调，实现新型城镇化建设新突破

推进新型城镇化提速提质，拉动投资，扩大消费。加快基础设施、公共服务向农村延伸覆盖，促进城乡协调发展。

促进农业转移人口市民化。以建设国家新型城镇化综合试点城市为契机，深化户籍制度改革，实施居住证制度，推进转移人口与城镇居民享有同等权利和义务，配套推进农村产权制度改革，保障农民财产性权益，为农民进城敞开门、解开绳。全市常住人口城镇化率增长2个百分点以上。

加快现代城镇体系建设。搞好我市第四轮城市总规修编和土地利用总规调整完善，加快编制县域城市总体规划、村庄规划和城市近期建设规划。围绕打造区域性中心城市加快市城区建设，着力推进“五网四厂两道”基础设施建设，抓好综合管廊建设试点，积极推进市、县海绵城市试点项目建设。实施开州路北延、濮上路北延、振兴路北延等道路建设。加快城中村和棚户区改造步伐，完成市第二污水处理厂二期工程建设。加快文明路、干城、濮清等农贸市场标准化改造和建设，推进文化宫广场地下停车场等6个公共停车场建设，建设城市公共自行车系统一期工程。开工建设新阳光大厦、市民综合活动中心、科技档案博物馆等项目，加快市法院审判法庭、市检察院办案技术用房及警示教育基地建设。做好建设用地供给管理，重点抓好两湖连接渠北侧区域、长庆片区等6个土地一级开发项目。加快县域中心城市和小城镇建设。完善基础设施，提升服务能力，打造宜居宜业县城。继续抓好33个建制镇综合提升，着力培育特色小城镇。加快数字化城市管理向智慧化升级，全面提升管理水平。巩固全国文明城市创建成果，塑造城市文化和城市精神，让城市散发文化的光束。

加快美丽乡村建设。以新理念引领农村新发展，创新机制，加大投入，点、线、面结合，综合整治村容村貌、路容路貌，着力补齐农村基础设施、社会管理、文化服务等方面的短板，努力开创美丽乡村建设新局面。实施“乡村畅通工程”，新建改造农村公路470公里。加强农村危房改造，持续推进农村人居环境综合整治，不断完善农村文体设施，切实抓好美丽乡村示范乡、示范村建设，让农村变得更美丽，让农民生活得更舒心。

（三）强化市场导向，实现现代农业发展新突破

加快转变农业发展方式，发展多种形式适度规模经营，促进农村三次产业融合发展，实现稳粮增收、提质增效。

提高粮食生产能力。落实藏粮于地、藏粮于技战略，抓好高标准粮田“百千万”工程，完成30万亩高标准粮田建设任务。推进渠村灌区、南小堤灌区、彭楼灌区、辛杨干渠续建配套与节水改造工程，加快小型农田水利重点县、金堤河防洪除涝工程建设。以实施引黄入冀补淀工程为契机，强化清丰县、南乐县农业灌溉能力建设。粮食总产稳定在50亿斤以上。

加快农业结构调整。实施都市生态农业发展工程，着力推进市城区半小时生态农业圈和5个县级都市生态农业示范区建设，大力发展休闲观光、创意农业等新型业态，不断满足城乡居民多重消费需求。加快汇源肉羊养殖基地、屠宰加工、冷链物流一体化建设。推进河南永达肉鸡养殖、牧原100万头猪场等项目建设。加快现代农业园区建设，新建设施农业园区6个。扶持壮大农业产业化龙头企业，新增产值超亿元龙头企业4家，大力发展农业产业化集群，构建“公司+基地”、“市场+基地”的经营格局。

培育新型农业经营主体。积极培育种养大户、家庭农场、农民合作组织、专业公司等新型经营主体。大力发展机械化农业，提高农业生产效率。统筹各类教育资源，培育高素质职业农民队伍。加强农产品流通设施和市场建设，积极发展“互联网+农业”。

推进农村产权制度改革。基本完成农村土地承包经营权、宅基地使用权、集体建设用地使用权的确权颁证。做好濮阳县“两权”抵押贷款试点和华龙区农村集体经济组织股份制改革试点工作。设立农村土地流转服务中心，促进农业适度规模经营。采取支农专项中筹措资金与争取上级支持资金相结合的方式，建立农业信贷担保平台，支持农业发展。完善涉农保险制度，扩大现有险种覆盖面。

（四）顺应消费趋势，实现服务业发展新突破

切实把发展服务业摆在更加突出的位置，顺应消费趋势，跟进消费热点，抓好六大重点领域，加快释放发展潜力。

大力发展现代物流业。围绕建设区域性物流中心，加快建设中原煤炭储配中心、濮阳市石化能源物流仓储基地、豫鲁商贸物流港、豫北商贸物流园、海王药械阳光集中配送中心、台前县电商物流中心等项目。

加快发展金融业。争取郑州银行、兴业银行、交通银行等来濮设立分支机构。加快农信社改制农商行步伐，争取濮阳农村商业银行、范县农村商业银行挂牌开业，濮阳县、清丰县、南乐县农信社达到组建农商行标准。督导各村镇银行加大乡（镇）机构建设力度。支持互联网金融、金融租赁业发展。

大力发展商贸服务业。围绕把濮阳建成冀鲁豫三省交会区域性重要商贸城市这一定位，科学编制发展规划，加快市场建设，培育壮大龙头企业，积极推进油田商贸中心、奥特莱斯商业综合体、怡亚通物流产业园等招商项目和正大五洲商贸中心、上亿广场等重点项目建设。推动传统企业转型升级，支持电商企业发展壮大，大力发展商贸服务新业态。重点抓好华龙区、开发区、台前县电子商务产业园建设，发挥电商企业集聚效应。推进跨境电子商务示范区创建工作。

加快发展文化旅游产业。规划建设濮阳县文化产业带，重点抓好中华第一龙遗址公园暨上古文化博物馆、五代城墙保护、澶州州署、明清古十字街保护提升等项目，叫响“中华龙都”、“华夏帝都”品牌。完善提升濮阳国际杂技文化产业园、东北庄原生态杂技文化旅游园区，加快建设杂技艺术学校，叫响“杂技之乡”品牌。以毛楼旅游景区、郑板桥纪念馆、将军渡提升和渠村黄河大闸景观改造为重点，打造黄河生态文化休闲旅游带。推进龙湖沿岸综合开发、示范区滨河特色街、金堤河国家湿地公园二期、引黄入冀补淀生态景观长廊等项目，打造养生、休闲、旅游目的地。依托单拐、颜村铺等革命旧址，大力发展红色旅游。

加快发展信息服务和体育健康养老产业。加强信息网络基础设施建设，推进“三网”融合发展，推动城乡上网普及提速，优化4G网络，实施城区无线局域网全覆盖工程。加快智慧城市建设，谋划建设大数据智慧生态园。发挥全国乒乓球训练基地的品牌和人才资源优势，发展乒乓体育产业。抓好濮阳医养中心、康复医院等项目建设。

促进房地产业健康发展。完善落实“房地产新政”，抓好对特定群体购房补贴。创新金融产品，支持农民进城购房；成立住房担保机构，为农民进城购房提供担保。引导房地产开发企业合理降价，降低农民进城购房成本。加大公积金归集扩面力度，将有稳定劳动关系的常住人口纳入覆盖范围。建立购租并举住房制度，培育发展住房租赁市场，公租房新增需求主要通过货币化补贴、由政府购买或长期租赁存量商品房解决。落实省棚户区货币化安置政策，提高棚改货币化安置率。优化住房供给结构，满足不同层次居民需求，促进房地产市场持续健康发展。

（五）厚植发展优势，提升区域竞争力

对接国家、省投资政策，谋划包装实施一批重大项目，拉动增长，增添后劲。着力抓好341个省市重点项目，年内完成投资826.33亿元。

加快完善综合交通体系。要全力以赴，确保年内开工建设郑濮济高铁，争取京九客运专线靠近濮阳城区。尽快开通晋豫鲁铁路濮阳段客运。谋划推进濮阳通用机场建设。加快台辉高速豫鲁界至范县段建设，积极推进郑州经滑县至濮阳高速、濮阳至商城高速等前期工作，构建“三纵三横”高速公路框架。加快干线公路建设，推进G341濮阳段等干线公路升级改造，构筑外通内联的综合交通体系，使濮阳人流、物流更加畅通便捷。

加快资源能源项目建设。开工建设文23战略储气库，推进新粤浙输气管道豫鲁支线、濮范台输气管道、中原至鹤壁输气管道等项目建设。推进日濮洛输油管线和范县至浚县成品油管线项目建设。加快豫能2×60万千瓦机组建设，开工建设保利协鑫电力清洁能源、盛润集团背压机组项目，推进台前2×100万千瓦机组、宏业生物质热电等项目。推进引黄入冀补淀工程、台前引黄调蓄水库等项目建设，形成资源能源富集地。

加快发展载体建设。产业集聚区要围绕主导产业，完善基础设施，加快公共研发、投融资、检验检测、物流会展、信息服务、市场交易平台建设，提升生产性和生活性服务功能，构建良好的产业生态圈。强化产城互动，推进城镇公共服务设施向产业集聚区延伸覆盖。加快集聚区内村庄整合迁建。加快构建与乡镇行政区域管理套合的管理体制。加快服务业“两区”建设，统筹推进路、电、水、讯等基础设施建设，完善综合配套服务设施，抓好项目引进和入驻，打造服务业发展高地。

加快人力资源开发。实施高层次人才引进工程，通过专业培养、实践锻炼、多元引入、柔性引进等方式方法，建立一支创新型人才队伍。深入实施全民技能振兴工程和职教攻坚工程，充分发挥职业技术学院、技师学院等各类职业教育机构的作用，形成一支技能型劳动大军。强化高端培训，着力锻造一支理念新、素质高、能力强的企业家队伍。加强智库建设，为濮阳发展提供人才保障和智力支持。

（六）突出改革创新，激发发展新动力

全面深化改革。认真落实中央和省、市委深化改革各项部署，推出一批具有重大牵引作用的改革举措。

深化投融资体制改革。加快投融资平台建设，力争将市投资集团资产规模增扩至200亿元以上，建立健全市、县（区）母子公司体制，提高投融资能力。改革政府投资项目机制，所有政府投资项目，均由政府投融资平台作为投资主体，各部门只履行监督管理和项目推进职能。运用多种融资工具，争取在发行私募债、项目收益债、建设专项债等方面取得新突破，实现融资50亿元以上。充分发挥龙都产业基金作用，加大对优质企业和上市后备企业资金扶持力度。积极推进中小企业发展基金设立工作，重点支持初创期、成长期中小企业发展。设立规模1.2亿元的现代农业发展基金，重点支持农业重点产业和特色产业发展。设立规模1.5亿元的科技成果转化及专利技术产业化发展基金，对种子期、初创期科技型企业及技术创新项目进行投资。做好国家专项建设基金和国家专项债券申报对接，争取更多项目纳入国家三年滚动投资计划或发行债券盘子。与浦发银行、豫资公司合作，设立濮阳浦银豫资PPP城市发展基金，规模100亿元，一期10亿元。充实市级PPP项目库，吸引社会资本参与基础设施和公共服务项目建设。抓好市担保集团的规范管理和运作，扩大助保贷、过桥资金规模，破解企业融资担保难题。实行存贷挂钩，强化激励，引导各金融机构最大限度地扩大信贷投放，新增贷款85亿元。加大资本市场融资力度，鼓励引导企业上市直接融资，推动天地人环保、佳化能源、巨烽能源等5家企业在新三板挂牌。引导支持企业通过发行企业债、产（股）权市场交易等多种方式融资，年内直接融资100亿元以上，增长20%。加强金融监测预警，切实防范化解金融风险。

加大国企改革力度。重点推进31户国有企业改革。引进央企、省企、民企参与国企改革重组，发展混合所有制经济。用好去产能政策，分类指导，妥善处置“僵尸企业”。完善体制机制，逐步实现各类经营性国有资产集中统一监管。

深化商事制度改革，全面落实“三证合一、一照一码”，推进电子营业执照和全程电子化登记，实现工商注册便利化。深化财税体制改革，落实营改增扩围、消费税改革和资源税费改革政策，推进国税、地税征管体制综合改革。实行中期财政规划管理改革，提高财政资金使用效率。深化医药卫生体制改革，抓好城市公立医院改革试点和县级公立医院综合改革。统筹推进教育、科技、环境保护等领域改革，不断释放改革红利。

实施创新驱动发展。将创新摆在发展全局的核心位置，聚合创新要素，提高创新能力，激发发展新动能。

发挥企业在创新中的主体作用。强化合作创新，构建以我市骨干企业为龙头，其他企业、高校、科研院所共同参与的产业技术创新战略联盟，搭建引领产业发展的高端创新平台。鼓励借智创新，支持企业追踪产业发展技术前沿，引进先进技术，在本地实现产业化。支持自主创新，年内新建省级工程技术研究中心3家、市级工程技术研究中心10家、市级重点实验室3个。新增高新技术企业3家、专利技术产业化示范企业5家、省节能减排科技创新示范企业2家、市节能减排科技创新示范企业4家。

汇聚创新资源。加强产学研合作，围绕重点产业，建设一批协同创新中心，实施一批协同创新重大项目。推进产业集聚区产业技术研究院建设，新建产业技术研究院5家，争取落户一批研发基地、中试基地，以创新引领产业集聚发展。推进国家知识产权试点城市建设，实施好专利技术产业化示范工程、知识产权优势培育工程。强化政府引领扶持，鼓励发展众创、众包、众扶、众筹空间，年内新认定市级众创空间10家。大力实施大众创业、万众创新工程，最大程度地释放全社会创新潜力。

以信息化深度融合推动创新。深入实施“互联网+”行动计划，以现代信息技术重构企业产业链，推动企业由单纯制造向服务型制造模式转变。新增“两化”融合示范企业5家。对接“中国制造2025”，重点支持贝英智能制造、华南重工高压电子及智能控制系统等项目，推进传统制造向智能制造演进。

（七）着力开放发展，增强发展新活力

发挥区位优势，对接国家和省重大战略，利用好国际国内两个市场、两种资源，融入经济大循环，向开放要活力。

拓展发展新空间。充分发挥晋豫鲁铁路联通港口的作用，找准我市对接“一带一路”战略和郑州航空港经济综合实验区的定位，打造节点城市，构建空港、陆港、海港联运格局，形成豫北出海大通道。要加快濮阳商检办事处业务中心建设，积极争取设立海关分支机构，先行启动海关保税物流中心建设，以郑州航空港申报自贸区为契机，谋划建设濮阳自贸区片区。加快走出去步伐，与外部需求对接，在更广范围内实现资源要素的优化配置，打开发展新空间。

实现招商引资新突破。坚持完善产业招商机制，突出招商重点，务求取得实效。突出招大引强，实施集群招商、精准招商，重点引进世界500强、国内领军企业等重大项目。突出战略重组，对接双方需求，力争在化工、装备制造、汽配加工等领域实现突破。突出创新引进，把握我市产业升级方向，跟踪技术前沿，对接先进企业，把引项目和引智引技结合起来，争取引进一批技术领先项目。突出项目落地，抓好签约项目的跟踪问效，责任到人，争取早日开工建设。要强化招商引资平台建设，提高服务水平，创造良好的营商环境。

（八）坚持绿色发展，建设生态文明

坚持治理、预防、建设、管理并重，下大决心，硬起手腕，采取得力措施，切实改善生态环境，努力实现绿色发展。

强化污染治理。落实最严格的耕地保护和环境保护制度，深入实施“蓝天工程”、“碧水工程”和“乡村清洁工程”。加大大气污染治理力度，实行控油、控煤、控尘、控车、控污“五控并举”，突出做好工业大气污染防治，淘汰和升级改造不符合要求的燃煤锅炉，切实抓好机动车污染控制和油品质量提升，加强建筑工地及道路交通扬尘治理，做好农作物秸秆禁烧工作，完善应急预警机制，遏制雾霾天气高发态势。加强河道治理，搞好城市雨污水分离，严禁工业、生活污水乱排乱放。深入推进乡村清洁工程，强化农业面源污染治理。加强空气、水质地质环境检测，及时发现和解决突出问题，不断改善环境质量。

强化源头预防。支持企业实施节能环保技改。完善污染企业退出机制，严禁新上环保不达标项目。加快对化工园区的循环化改造，转变能源利用方式，提升资源利用效率，减少污染物排放。规划建设静脉产业园，建设城区生活垃圾焚烧发电、建筑垃圾再加工利用、医疗垃圾处置等项目，实现垃圾和废弃物的循环利用和无害化处理，全面提高节能、降耗、减排水平。

强化监督管理。建立完善生态保护红线管理制度和污染防治联动机制，建立健全企业排放许可、评价考核与责任追究制度。建立企业排放在线监测体系，严惩偷排超排行为。强化依法治理，保障环境质量的明显好转。

强化生态建设。努力创建国家森林城市，实施廊道绿化、农田林网、湿地郊野公园等重点工程，推进苗木花卉和经济林两大基地建设，完成生态工程造林6万亩以上。加强全域河流生态保护，建设健康水系。推进生态城市建设，做活水、做足绿。推进老马颊河等河道综合治理，实施市城区水系连通工程，建设引黄入冀补淀生态景观长廊，让城市之水活起来、美起来；加快龙山、水库绿化建设，提升环城林系和城市道路绿化水平，规划建设一批城市生态小景。加大县城、小城镇生态建设，营造绿色空间、宜居环境。

（九）突出精准脱贫，全力打好脱贫攻坚战

我市现有贫困人口19.1万人，脱贫攻坚任务艰巨。要切实把脱贫攻坚摆在全面建成小康社会的战略位置，全力抓好，务求实效。

深入贯彻精准扶贫、精准脱贫基本方略，多措并举抓脱贫。一是抓好转移就业脱贫。整合培训资源，开展订单、定向培训，提高就业创业能力。完善劳务对接机制，促进贫困人口稳定就业。二是抓好发展产业脱贫。支持贫困区域因地制宜发展特色产业，扶持建设一批贫困人口参与度高的增收项目，为贫困群众脱贫致富提供产业支撑。三是抓好教育脱贫。加大贫困家庭子女教育扶持力度，免除建档立卡困难家庭普通高中学生学杂费，对贫困家庭大学生实施救助，以教育助推脱贫。四是抓好救助脱贫。探索建立城乡大病保险向贫困对象、高额医疗费用患者倾斜机制，防止因病致贫、因病返贫。五是抓好兜底脱贫。完善农村最低生活保障制度，对完全或部分丧失劳动能力的贫困人口应保尽保。六是抓好滩区迁建试点。切实抓好范县张庄乡、陈庄镇搬迁试点迁建工作，尽快实现搬迁入住。积极与省搞好对接，启动第二批迁建试点工作。

完善长效机制，实行台账管理，注重跟踪问效，严格问责，扎实推进，确保年内实现精准脱贫6.13万人。

（十）加强社会建设，增进人民福祉

保民生、兜底线，更加关注低收入群众生活，着力实施好十项重点民生工程，让人民群众有更多的获得感。

强化基本民生保障。完善就业创业服务体系，城镇新增就业4万人，失业人员再就业1.8万人，就业困难人员再就业5500人，动态消除“零就业家庭”。发放创业担保贷款4亿元，扶持带动1.5万人创业就业。提高社会保障水平，城乡低保财政补差水平月人均分别提高15元、12元，农村五保对象年集中供养、分散供养标准分别提高到人均4000元、3000元。健全企业工资集体协商、正常增长和支付保障机制，继续提高企业退休人员基本养老金。依法治理拖欠农民工工资问题，切实维护其合法权益。深化机关事业单位养老保险制度改革，适当提高机关事业单位人员基本工资，落实县以下机关公务员职务与职级并行制度和乡镇工作补贴。建立统一的城乡居民基本医疗保险制度，推进省内异地就医即时结算，全面实施城镇居民大病保险，为3.5万农村妇女免费实施“两癌”筛查。建设15个社区日间照料中心、600个农村互助幸福院，开工建设市综合养老服务中心、市残疾人综合服务中心，加快建设市烈士陵园。新建、购买各类保障性住房1.2万套。提高城市饮水质量，让居民喝上纯净的丹江水。

优先发展教育事业。启动新一轮农村义务教育学校布局调整。改善125所义务教育薄弱学校办学条件，对53所农村中小学校舍进行改造，新建改建市城区学校10所。实施好乡村教师支持计划，提高乡村教师生活待遇，畅通城镇优秀教师到乡村学校任教通道，促进城乡教师资源合理配置。实施第二期学前教育三年行动计划，全市新建30所、改扩建76所幼儿园。落实普惠性中等职业教育政策。办好特殊教育。支持河南大学濮阳工学院扩大规模。加快市委党校新校区建设。

着力加强文化建设。积极践行社会主义核心价值观，深入开展精神文明建设，强化舆论引导，讲好濮阳故事，传播汇聚正能量。完善公共文化服务体系，建成投用市图书馆新馆，市城区建设20个流动书屋，建设书香濮阳。建设400个村级综合公共文化服务中心。推进文化、科技、金融、旅游、互联网融合发展。深化文化体制改革，推动传统媒体和新兴媒体融合发展，加快国有经营性文化单位转企改制，深化公共文化服务单位内部管理机制改革。积极发展体育产业，加强体育基础设施建设，广泛开展全民健身活动，提高竞技体育发展水平。

全面改善医疗卫生条件。持续开展爱国卫生运动，推进城乡环境卫生整洁行动。大力发展医疗健康产业，完善提高市、县、乡、村四级卫生服务网络，努力构筑冀鲁豫三省交界高端医疗服务中心。加快市人民医院病房楼、市红十字医院康复病房楼等项目建设。新建提升14所社区卫生服务中心，改扩建20所乡（镇）卫生院、新建改造260所村卫生室。全面落实两孩政策。

完善社会治理。完善城市社区和农村基层服务体系，提升社会治理水平。强化食品安全监管，争创全国食品安全示范市。加大安全生产监管力度，坚决遏制重特大事故发生。加强信访维稳工作，排查化解社会矛盾，做好特殊人群的教育管理和服务帮扶，加强宣传教育，远离非法集资，防范和化解各类社会风险。加强社会治安综合治理，强化应急管理体系建设，完善立体化社会治安防控体系，完善反恐工作机制，坚决打击违法犯罪活动，打造平安濮阳、法治濮阳。

加强国防教育和国防后备力量建设，支持驻濮解放军、武警消防部队、预备役部队、民兵和人民防空建设，深入开展双拥共建，巩固军政军民团结。认真听取民主党派、工商联、无党派人士意见，支持工会、共青团、妇联开展工作。做好民族宗教工作。统筹做好统计、发展研究、外事侨务、机关事务、驻外联络、残疾人服务、史志、档案、防震减灾、气象、邮政、通信等工作。

四、加强政府自身建设

推进依法行政。加快法治政府建设，严格依照法定权限和程序管理经济社会事务。健全依法决策机制，坚持公众参与、专家论证、风险评估、合法性审查、集体决定等重大行政决策程序，确保决策民主科学合法。推进服务型行政执法，加强行政复议和行政执法监督，严格行政执法责任制，严格规范公正文明执法。加强法治宣传教育和依法治理，启动“七五”普法工作。严格执行人大及其常委会的决议、决定，自觉接受人大监督、政协监督、社会监督和舆论监督。完善政府内部层级监督，强化监察、审计等专门监督，让行政权力在法治轨道和阳光下运行。

提升行政效能。加快转变政府职能，深化放、管、服改革，再清理压减一批行政审批事项、审批中介服务事项。落实权责清单制度，实行动态管理，加强和改善事中事后监管。推行行政服务标准化建设，推广“互联网+政务服务”。深化公共资源交易改革，建立统一规范的公共资源交易平台。推进懒政怠政为官不为专项治理，整治行政不作为、慢作为现象，消除 “中梗阻”，打通政策落实“最后一公里”。强化激励约束，健全岗位履职责任制和激励机制，完善督查和纠错问责机制，形成层层负责、人人担当的强大合力。

加强廉政建设。坚持纪严于法、纪在法前，严明政治纪律和政治规矩，履行主体责任和“一岗双责”，全面落实党风廉政建设责任制，强化惩防体系建设。加强党规政纪教育，严格执行中央八项规定精神和省市实施意见，模范遵守廉政准则和各项规定，加大案件查办力度，保持反腐高压态势，坚决查处不正之风，弘扬清风正气，建设廉洁政府。

各位代表！

致力濮阳新一轮赶超发展，我们责无旁贷；让人民生活得更加幸福，我们孜孜以求。让我们紧密团结在以习近平同志为总书记的党中央周围，在省委、省政府和市委的坚强领导下，凝心聚力，砥砺奋进，为建设富裕文明和谐美丽新濮阳、全面建成小康社会做出新的更大贡献！
